# Typhoid Fever in Alabama

**Published:** 1850-02

**Authors:** 


TYPHOID FEVER IN ALABAMA.

The New Orleans Medical and Surgical Journal, for January, contains two excellent letters on the existence of typhoid fever in Alabama, —one by W. B. Johnson, M. D., on the diseases of Marion, Alabama, the other by C. J. Clark, M. D., of Jacksonville, Alabama. It seems to us that these two writers perfectly understand what they are describing, and they fully prove the existence of typhoid fever in those regions of Alabama, in which they respectively practice. Both writers show a discriminating judgment, and we rely implicitly on their statements.—Dr. Clark, especially, sustains himself with perfect fullness, and if the cases he describes are not instances of typhoid fever, nothing of the kind has ever been seen. They resemble that disease as it has been depicted by Louis, Gerhard, and Bartlett, three names that constitute a tower of strength, of an unimpregnable character, on the subject of typhoid fever. We have not often seen a better medical letter than that of Dr. Clark's. It is full, clear, and philosophical.

In allusion to a former letter, on the existence of typhoid fever, Dr. Clark says: "In my letter to Dr. Lewis, speaking of the prevalence of this fever, in this year, (1840), I said it attacked '*indiscriminately all ages*, without regard to sex or color.' This expression was carelessly made, and put into that particular language rather to 'round a period,' than express accurately a fact. The idea intended to be conveyed was, that I had seen typhoid fever occurring in children, adults and old persons, in males and females, and that both blacks and whites had been subject to it." In reference to the treatment received by him for writing this careless sentence, Dr. Clark says: "This unfortunate sentence was quoted and requoted," by a reviewer, "to invalidate the writer's testimony as to the existence of typhoid fever in this part of the State, in a manner that might do well enough for a distressed critic, but not for the candid inquirer after truth." That term, "distressed critic," exactly expresses the idea; its author must have served a time at the art of driving nails, from the dexterity with which he has driven this one home to its place.

We have seldom read medical articles of more interest than the letters to which we have referred. They were conceived in an admirable spirit, and executed with fidelity.

